# A rapidly deployable individualized system for augmenting ventilator capacity

**DOI:** 10.1126/scitranslmed.abb9401

**Published:** 2020-05-18

**Authors:** Shriya Srinivasan, Khalil B Ramadi, Francesco Vicario, Declan Gwynne, Alison Hayward, David Lagier, Robert Langer, Joseph J. Frassica, Rebecca M. Baron, Giovanni Traverso

**Affiliations:** 1 Department of Mechanical Engineering, Massachusetts Institute of Technology, Cambridge, MA 02139, USA.; 2 Division of Gastroenterology, Hepatology and Endoscopy, Brigham and Women’s Hospital, Harvard Medical School, Boston, MA 02115, USA.; 3 David H. Koch Institute for Integrative Cancer Research, Massachusetts Institute of Technology, Cambridge, MA 02139, USA.; 4 Division of Comparative Medicine, Massachusetts Institute of Technology, Cambridge, MA 02139, USA.; 5 Department of Chemical Engineering, Massachusetts Institute of Technology, Cambridge, MA 02139, USA.; 6 Institute for Medical Engineering and Science, Massachusetts Institute of Technology, Cambridge, MA 02139, USA.; 7 Department of Anesthesia, Critical Care and Pain Medicine, Massachusetts General Hospital and Harvard Medical School, Boston, MA, 02139.; 8 Philips Research North America, Cambridge, MA 02141, USA.; 9 Institute for Medical Engineering and Science, Massachusetts Institute of Technology, Cambridge, MA 02139, USA.; 10 Division of Pulmonary and Critical Care Medicine, Brigham and Women’s Hospital, Harvard Medical School, Boston, MA 02115, USA.

## Abstract

Strategies to split ventilators to support multiple patients requiring ventilatory support have been proposed and used in emergency cases in which shortages of ventilators cannot otherwise be remedied by production or procurement strategies. However, the current approaches to ventilator sharing lack the ability to individualize ventilation to each patient, measure pulmonary mechanics, and accommodate rebalancing of the airflow when one patient improves or deteriorates, posing safety concerns to patients. Potential cross-contamination, lack of alarms, insufficient monitoring, and inability to adapt to sudden changes in patient status have prevented widespread acceptance of ventilator sharing. We have developed an individualized system for augmenting ventilator efficacy (iSAVE) as a rapidly deployable platform that uses a single ventilator to simultaneously and more safely support two subjects. The iSAVE enables subject-specific volume and pressure control and the rebalancing of ventilation in response to improvement or deterioration in an individual’s respiratory status. The iSAVE incorporates mechanisms to measure pulmonary mechanics, mitigate cross-contamination and backflow, and accommodate sudden flow changes due to subject interdependencies within the respiratory circuit. We demonstrate these capacities through validation using closed- and open-circuit ventilators on linear test lungs. We show that the iSAVE can temporarily ventilate two pigs on one ventilator as efficaciously as each pig on its own ventilator. By leveraging off-the-shelf medical components, the iSAVE could rapidly expand the ventilation capacity of healthcare facilities during emergency situations such as pandemics.

## INTRODUCTION

Ventilators are vital equipment for critical-care conditions, surgical interventions, and procedures requiring anesthesia. Ventilators assist with respiration by moving air into and out of the lungs and are extensively used in the treatment of patients with acute respiratory distress syndrome (ARDS) and respiratory failure. Pandemics of infections that cause severe respiratory dysfunction can lead to a rapid increase in the volume of patients requiring ventilation, exceeding the number of ventilators available in healthcare facilities and necessitating difficult triage decisions (*1*, *2*). The most recent publicly available data (2010) reports that about 62,000 full-featured mechanical ventilators are available within the United States, with an additional 12,700 stockpiled in the Centers for Disease Control and Prevention strategic national stockpile (SNS) (*1*). Based on quantified disruptions in supply chain, recent coronavirus disease 2019 (COVID-19) hospitalization rates, and predictive models, estimates of the shortfall of ventilators within the U.S. range between 45,000 - 160,000 (*1*–*4*). Globally, the World Health Organization (WHO) estimates that fewer than 2,000 ventilators are available across the 41 African countries (*5*) to support a population of 1.2 billion people. Coupled with high population density and relative poverty, pandemics can make the procurement, building, or buying of new, emergency ventilators challenging for some countries. Low-cost and emergency ventilators have been developed to address the cost barrier associated with the acquisition of new ventilators at scale. However, their manufacturing and deployment relies on supply, assembly, and distribution chains which can be disrupted by public health crises involving lockdowns and import/export restrictions. The use of new ventilator designs has also raised safety concerns, as clinical staff would need to operate unfamiliar technology. Given both supply and implementation hurdles that may delay rapid deployment of low-cost ventilators, other strategies warrant consideration.

Ventilator sharing, or dividing the airflow from one ventilator among multiple patients, has been previously performed in a few emergency cases (*6*). By using readily available tubing and ventilatory equipment, ventilator sharing can be immediately implemented to expand the capacity of existing ventilators with which clinicians are familiar. In previously proposed configurations, multiplexing ventilation involved connecting multiple outflow tracts to the ventilator to divide flow amongst patients (*6*–*9*) wherein the compliance (*C*) and resistance (*R*) of each patient’s pulmonary system became part of the same circuit and drove the balance of airflow. This patient interdependence poses various safety concerns: (i) Independent control of volume and pressure to each patient is not possible, which is important for lung-protective ventilation and the standard of care for ARDS; (ii) alarm monitoring becomes challenging due to the complex circuit configuration; (iii) sudden events such as pneumothorax, tube occlusion, or disconnection of an endotracheal tube, causes potentially harmful rebalancing of ventilation; and (iv) changes in one patient’s condition (clinical improvement or deterioration) results in an automatic change to ventilation of other patients. Other practical challenges include monitoring, routine measurement of pulmonary mechanics, overcoming ventilator self-tests/calibration, and the risk of exposure due to a break in the circuit that aerosolizes respiratory-borne infectious agents when adding and removing patients. For these reasons, medical associations including the American Association for Respiratory Care (AARC) issued a joint statement explicitly advising clinicians against the sharing of mechanical ventilators with current approaches (*10*).

To expand ventilator capacity while incorporating the constraints associated with supply chain/distribution limitations, we engineered the individualized system for augmenting ventilator efficacy (iSAVE), which repurposes medical-grade valves, sensors, and filters to allow a single ventilator to provide personalized volume and pressure support to at least two patients. The use of repurposed components can enhance ventilator capacity independent of supply chain limitations. Here, we describe the design and validation of the iSAVE through benchtop and in vivo tests sharing a single ventilator among two pigs. We hypothesized that our system could maintain specified ventilation parameters to each subject amidst static and dynamic changes in resistance and compliance. We simulated clinical scenarios, focusing on those relevant to the management of ARDS, and validated the safety mechanisms of the system. Leveraging off-the-shelf components, the iSAVE can rapidly expand existing ventilation capacity of healthcare facilities.

## RESULTS

### Design of the iSAVE

**Fig. 1:**
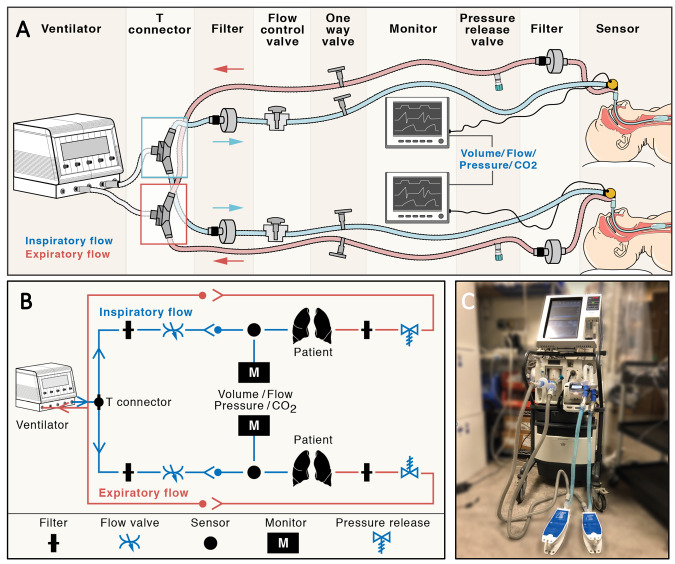
Design of the individualized system for augmenting ventilation efficacy (iSAVE). (**A**) Schematic of iSAVE setup on a closed-circuit ventilator for simultaneous ventilation of two patients. (**B**) Circuit diagram of iSAVE for closed-circuit ventilation. (**C**) Photograph of iSAVE connected to a Puritan Bennet 840 ICU ventilator and two test lungs.

The iSAVE uses a series of valves and flow regulators in parallel limbs to effectively maintain the desired tidal volume (*V*_T_) and positive end-expiratory pressure (PEEP) for each patient (Fig. 1, tables S1 and S2) under volume control mode. In closed-circuit ventilators (Fig. 1), Y or T connectors are used to multiplex individual inspiratory channels for each patient. Each inspiratory channel consists of a filter, flow control valve, a one-way flow valve, and standard sensors (pressure, flow, capnostat) in series. The expiratory limb consists of a filter, pressure release valve, and one-way valve prior to connection to Y or T connectors which are routed back to the ventilator. The iSAVE can be configured to both open-circuit ventilators (which consist of only an inspiratory limb with passive expiration) and closed-circuit ventilators (which possess inspiratory and expiratory limbs). Open-circuit ventilators can be used with the same circuit shown in Fig. 1, except that the expiratory limb would be connected to a Whisper Swivel (Philips Respironics) exhalation adaptor or equivalent valve for the expiratory flow port. We used positive expiratory pressure (PEP) threshold devices as one-way valves, set to their highest setting, because these are readily available in hospitals. See tables S1 and S2 for a full list of supplies.

The flow control valve is used to allocate the appropriate *V*_T_ to each patient. Filters on each limb mitigate cross-contamination between individual patient circuits and filter expired gas before release into the room through the pressure release valve, thus limiting pathogen exposure to healthcare workers. The one-way valves prevent backflow and mitigate over-distention in cases of rapid flow change. PEEP valves enable the individualized control of PEEP for each patient and function as a pop-off valves to release excess pressure. Pressure, flow, and CO_2_ sensors are positioned on the patient Y piece and data, visualized on a separate patient monitor, is used for setup, titration, and monitoring. During initial setup, the respiratory rate (RR), PEEP, FiO_2_ (fraction of inspired oxygen), inspiration:expiration (I:E) ratio, and the sum of the *V*_T_ for each patients is set on the ventilator under volume control mode. Then, the flow control and PEEP valves can be titrated to individualize *V*_T_ and PEEP. The exhaled volume and minute ventilation alarms on the ventilator are set according to the sum of the exhaled tidal volumes from both patients and enable the ventilator to alarm in response to sudden change in either patient’s status (shunt, occlusion, or disconnection of the endotracheal tube). Through this design, the iSAVE overcomes many of the aforementioned challenges of splitting ventilation (Table 1).

**Table 1:** Key challenges in splitting ventilation. A comparison of the capabilities of existing splitting mechanisms and iSAVE. *See fig. S9 for details regarding the rerouting of standard sensing metrics required for ventilator calibration and self-tests. PEEP, positive end-expiratory pressure; FiO_2_, fraction of inspired oxygen; Δ*C*, change in compliance; Δ*R*, change in resistance; *P*_plat_, plateau pressure.

**Concern**	**Uniform splitting** **(pressure control mode)**		**iSAVE** **(volume control mode)**	
Individualized management of ventilation				
- PEEP	x	Shared between patients	o	Individualized to each patient	
- Tidal volume	x	Shared between patients	o	Individualized to each patient
- FiO_2_, respiratory rate	x	Shared between patients	x	Shared between patients
- Alarms	x	Changes to one patient’s status may not result in main ventilator alarm	o	Changes to one patient’s status will cause main ventilator to alarm. Mechanical components to provide auditory alarms can be incorporated
Sudden changes to patient status can cause damaging rebalancing of airflow to other patient(s) toward most compliant lungs	x	Ventilation cannot be quickly adjusted	o	Can be managed by titrating flow control valves. One-way valves prevent backflow. Pressure release valves prevent excess pressure delivery
Improvement or deterioration of one patient (Δ*C*, Δ*R*) will automatically rebalance airflow, potentially harming other patient(s)	x	Ventilation cannot be individually rebalanced. Patients would need to be re-matched as they improve/deteriorate	o	Desired ventilation for each patient can be achieved through valve adjustment, allowing patients to improve/deteriorate while remaining on the same system.
Abruptly removing patients requires breaking the circuit, causing aerosolization of the virus, exposing healthcare personnel	x	Individual patient circuits cannot be quickly removed from circuit	o	Individual patients can be quickly shunted/removed from the circuit. Inline filters limit aerosolization risk
Monitoring	x	Additional respiratory monitors and heightened clinical vigilance required	x	Additional respiratory monitors and heightened clinical vigilance required
Measurement of pulmonary mechanics	x	Shared between patients	o	*P*_plat_ can be measured using expiratory hold button. *C*, *R* can be computed for each patient
Ventilator calibration/self-test	x	Added circuit volume defeats the operational self‐test	o	Can be executed with modifications to circuit*
Triggering	x	Disabled. Patients will require sedation	x	Disabled. Patients will require sedation

### Benchtop testing of the iSAVE using linear test lungs

The iSAVE was connected to three common models of intensive care unit (ICU) ventilators (Hamilton, Puritan Bennett, and Philips) (fig. S1). Testing confirmed the circuit was generalizable and could be used to ventilate two linear test lungs.

#### Individualized management of ventilation and patient interdependence

**Fig. 2:**
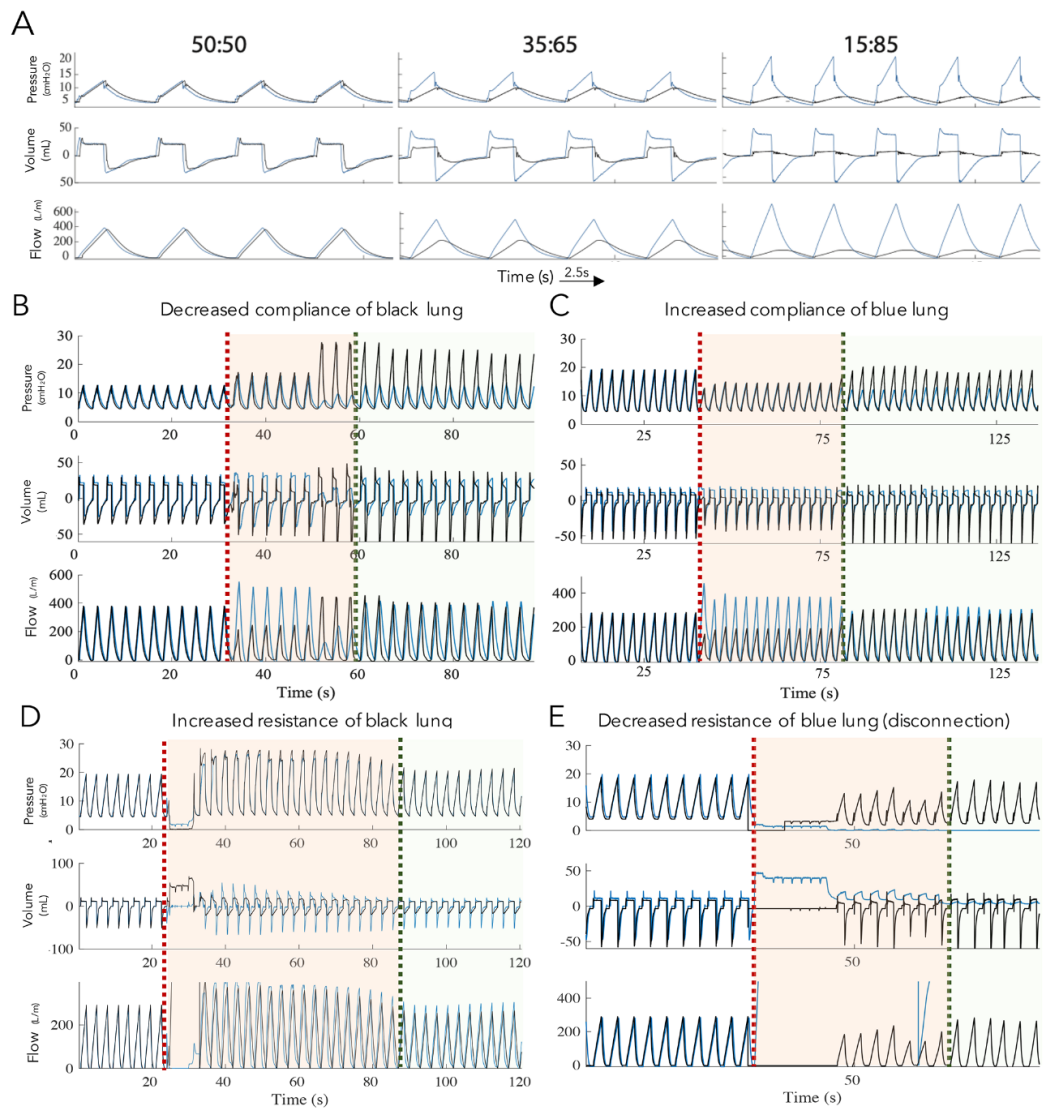
**Individualized ventilation and management of patient interdependence using artificial test lungs**. (**A**) Pressure, flow, and tidal volume waveforms illustrating three settings of differential tidal volume (*V*_T_) for two test lungs (blue, black) using closed-circuit ventilation. The ratio (50:50, 35:65, 15:85) refers to the *V*_T_ of the black:blue lungs. Pressure, volume, and flow in both lungs upon (**B)** decreased compliance in one lung (black) and (**C**) increased compliance in the other lung (blue). The orange dotted line indicates decrease or increase in compliance. The green dotted line indicates return of baseline ventilation parameters upon titration of the valves. Pressure, volume, and flow in both lungs upon (**D**) increased resistance in one lung (black) and (**E**) decreased resistance in the other lung (blue). Orange dotted line indicates increase or decrease in resistance. Green dotted line indicates return of baseline ventilation parameters upon titration of the valves. Waveforms from each lung are slightly offset to enable visualization.

We tested the ability of the iSAVE to meet the diverse *V*_T_ needs that may be presented by patients of varying sizes and respiratory mechanics. We first delivered *V*_T_ of 800 mL to two test lungs with healthy (*C* = 50 mL/cmH_2_O, *R* = 5 cmH_2_O/L/s) and diseased (*C* = 20 mL/cmH_2_O, *R* = 5 cmH_2_O/L/s) pulmonary characteristics. By titrating the flow control valve, we were able to achieve differential *V*_T_ spanning ratios from 50:50 to 15:85 (Fig. 2A). However, at ratios more disproportionate than 20:80, pressure exceeded 40 cmH_2_O, which is supratherapeutic.

#### Accommodating lung compliance and resistance changes

Respiratory mechanics for patients with ARDS can vary considerably and evolve rapidly throughout the course of disease and recovery. Assuming that patients are initially matched, clinical improvement or deterioration will yield mismatches in airflow, requiring individualized management for optimized therapy. We tested the ability of the iSAVE to compensate for static changes in compliance and resistance of one test lung while minimizing effects on the other lung. For all tests, we (i) measured the baseline ventilation values, (ii) performed an intervention to change compliance or resistance, (iii) noted any safety features or alarms that were activated, and (iv) titrated valves to restore ventilation to baseline (within 5% error).

We decreased the compliance of one of the test lungs (from *C* = 50 mL/cmH_2_O to *C* = 15 mL/cmH_2_O), simulating the parameters characteristic of ARDS (Fig. 2B). Flow was quickly diverted, resulting in a disproportionate volume being delivered to the healthy lung (*C* = 50 mL/cmH_2_O). The PEEP valve released excess volume during the period of titration, preventing overdistention of the healthy lung. The desired volume of 400 mL/lung was restored by titrating the flow control valve. We next began with two lungs simulating ARDS (*C* = 20 mL/cmH_2_O, *R* = 5 cmH_2_O/L/s) and increased the compliance of one lung (from *C* = 20 mL/cmH_2_O to *C* = 40 mL/cmH_2_O), which created a shift in the volumes delivered. Adjustment of the flow control valve restored the desired flow to both lungs (Fig. 2C).

We then performed ventilation (300 mL/lung) under high resistances, simulating the physiology characteristic of the comorbidities commonly associated with ARDS, including bacterial pneumonia, asthma, chronic obstructive pulmonary disease/emphysema, and presence of viscous airway secretions. With both lungs simulating ARDS (*C* = 20 mL/cmH_2_O, *R* = 5 cmH_2_O/L/s), the resistance of one lung was increased ten-fold (*R* = 5 cmH_2_O/L/s to *R* = 50 cmH_2_O/L/s), causing a drastic reduction in the flow to the lung. Titration of the valve along with an increase in the inspiratory time enabled the desired flow to be achieved while maintaining lower pressures (Fig. 2D). The aforementioned benchtop tests were also performed with an open-circuit ventilator, yielding similar results (figs. S2 to S4).

#### Managing abrupt changes in respiratory status and adjusting the number of subjects on the iSAVE

Whereas subacute changes in resistance and compliance can be accommodated, it is vital that the iSAVE enables alarms in response to acute changes for patient safety. The ventilator alarm was set to detect changes in the overall expiratory volume. We mechanically occluded tubing of one lung to simulate an instantaneous change in resistance, which created a reduction of flow in one channel and spike in pressures/volumes of the other (fig. S5). This successfully caused the ventilator alarm to activate instantaneously. We also simulated the loss of the endotracheal tube to one lung, resulting in a leak in the system and yielding minimized flow to the other lung. This activated the main ventilator leak alarm. We immediately closed the valve to the disconnected affected lung’s circuit, effectively closing the leak and removing the test lung from the circuit. We then titrated the other lung’s valve to deliver the desired volume (Fig. 2E). This process of shutting airflow to one segment of the circuit could be used in cases such as cardiac arrest or weaning from the ventilator to remove a patient from shared ventilation without leaking air and thereby mitigating potential aerosolization of infectious agents.

A practical challenge to be managed by this system is the addition of patients without excessive disruption to other patients. We simulated the addition of a second patient to a shared ventilator while minimizing deleterious effects to the original patient with a series of protocolized steps using artificial lungs (fig. S6).

#### Cross-contamination

It is critical that the iSAVE prevent cross-contamination under potentially turbulent or unusual airflow patterns caused by shared ventilation, particularly for use in patients with highly airborne and infectious pathogens. Contamination patterns were tested by nebulizing trypan blue into the airflow tract of one artificial lung and testing for contaminants in each segment of the circuit using a closed-circuit ventilator (fig. S7A). Even at unrealistic conditions yielding turbulent flows at high pressures [nebulization at 40 cmH_2_O, 10 min of continuous nebulization, 5 mL of nebulized particles, *V*_T_ = 300 mL, RR = 30 breaths per minute (bpm)], no cross contamination of filters was visually observed (fig. S7B) or detected in wipe tests of each segment of the circuit (fig. S7C).

#### Measuring pulmonary mechanics

With shared ventilation, pulmonary mechanics used to optimize ventilation become challenging to evaluate for each patient. The measurement of plateau pressure (*P*_plat_)–the pressure maintained during inspiration when flow is equal to zero and approximates the alveolar pressure – can be used to calculate the lungs’ *R* and *C*. On closed-circuit ventilators with iSAVE, the end-inspiratory hold feature can be used to yield the *P*_plat_. On open-circuit ventilators with iSAVE, an additional flow valve can be added to the expiratory channel and briefly closed at the end of inspiration to yield *P*_plat_. While connected to two artificial test lungs using a closed-circuit ventilator, we performed an inspiratory hold and visualized *P*_plat_. Because of the circuits’ interdependence, *P*_plat_ was uniform between both lungs; However, PEEP and tidal volumes differed. Thus, accurate compliances can be computed. In three separate trials, we simulated this procedure with artificial lungs of varying compliances. Computed compliances were ≤ 11% error of the set compliances (table S3).

### In vivo testing

#### Individualized management of ventilation and patient interdependence

To evaluate the practical management of rebalancing ventilation using the iSAVE when ventilating real lungs, which exhibit variable respiratory mechanics, we performed closed-circuit ventilation using a large animal (Yorkshire swine, 70 kg) alongside an artificial lung (fig. S8A). The pig possesses a lung capacity of 5 to 6 L, similar to human lungs, and serves as a translational preclinical model for this testing. We varied the lung mechanics of either the porcine or artificial lung (1 L capacity) to test the system performance, delivering *V*_T_ = 500 mL from the ventilator. The iSAVE delivered differential volumes to each channel. Ratios of 50:50, 40:60, and 30:70 are presented in fig. S8B.

#### Accommodation to static compliance changes and altered respiratory status

To evaluate the system’s response to changes in lung compliance, *V*_T_ was initially distributed from the ventilator equally to each channel (600 mL total, 300 mL per channel). The compliance of the artificial lung was decreased (from *C* = 120 mL/cmH_2_O to *C* = 60 mL/cmH_2_O), resulting in a greater allocation of volume (~100 mL) to the porcine lung (fig. S8C). By titrating the flow valve, *V*_T_ distribution was restored within 3 breaths.

During the course of recovery from ARDS, lung compliance increases–thus, we tested a range of such scenarios (*C* = 20, 50, 60, and 120 mL/cmH_2_O). To model an extreme case, we adjusted the artificial lung compliance from *C* =20 mL/cmH_2_O to *C* = 120 mL/cmH_2_O: iSAVE immediately diverted flow away from the higher resistance in the animal’s lungs toward the less-resistant artificial lung (fig. S8D). Valve adjustment restored the desired flow to the animal’s lungs and artificial lung. Throughout these tests, end tidal carbon dioxide (EtCO_2_) and oxygen saturation (SpO_2_) were maintained between 38-41 mmHg and 91-98%, respectively, at baseline and after titration.

After euthanasia, the animal’s lungs were filled with 750 mL of saline, effectively decreasing their compliance and resulting in a lower *V*_T_. With repeated breaths, flow was further diminished despite efforts by the automatic adjustments of the ventilator in increasing the pressure (fig. S8E). Titration of the flow valve enabled restoration of the desired volume. We also simulated scenarios such as tube clogging and aspiration, in which the endotracheal tube of the artificial lung was mechanically obstructed. This immediately increased flow to the animal by 30% (fig. S8F) and was quickly resolved by closing the valve to the artificial lung circuit and adjusting flow to the animal.

#### Validating iSAVE ventilation against standard ventilation

**Fig. 3:**
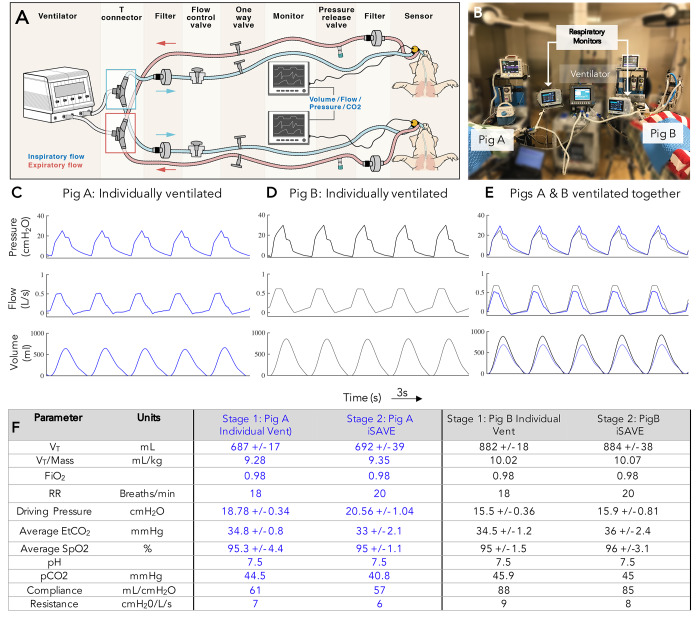
**Ventilation of two pigs on the iSAVE**. (**A**) Experimental setup for stage 2 and stage 3 of shared ventilation of pig A (74 kg) and pig B (88 kg) with iSAVE using closed-circuit ventilation. (**B**) Photograph of the experimental setup. Pressure, flow, and volume waveforms for (**C**) pig A ventilated individually (stage 1), (**D**) pig B ventilated individually (stage 1), and (**E**) pigs A and B ventilated together on the iSAVE (stage 3). (**F**) Table summarizing ventilatory and respiratory parameters and arterial blood gasses for (C-E). Mean ± SD was calculated from 300 breathing cycles. No significant differences were found between the individual and shared ventilation approaches (homoscedastic two-tailed *t* test, *P* > 0.05).

We then investigated whether the iSAVE could ventilate two large animals (Yorkshire swine, 74 kg and 88 kg) as adequately as ventilating each animal on its own closed-circuit ventilator (Fig. 3, A and B) in a 3-stage experiment. In stage 1, pigs were ventilated individually. Pig A required a *V*_T_ = 690 mL, whereas Pig B required a *V*_T_ = 880 mL. We ventilated each animal for about 45 min under stable EtCO_2_ (33-38 mmHg), SpO_2_ (93-99%), desired tidal volumes and arterial blood gasses (Fig. 3, C and D). In stage 2, we ventilated both animals on the same closed-circuit ventilator using iSAVE. Representative individual pressure, flow, and volume traces are provided in Fig. 3E and the means and standard deviations (SD) of 300 breathing cycles are provided in Fig. 3F. No significant differences were observed between stage 1 and stage 2 and all values were within the physiologically healthy range for swine. The difference in tidal volume between stage 1 and 2 was 5 mL for Pig A and 2 mL for Pig B. The difference in driving pressure between stages was 1.8 cmH_2_O for Pig A and 0.4 cmH_2_O for Pig B. Blood electrolytes, anion gap, glucose, blood urea nitrogen (BUN), hematocrit, and hemoglobin remained stable throughout (table S4). We measured the respiratory mechanics of both animals during both stages of ventilation. During shared ventilation, *P*_plat_ was simultaneously determined using the end-inspiratory hold feature for both animals. The computed compliances and resistances (based on the *P*_plat_ from the main ventilator) matched those displayed by the individual respiratory monitors.

**Fig. 4:**
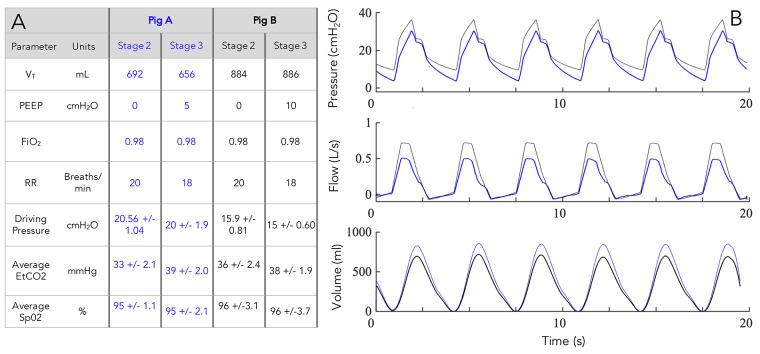
Differential tidal volume and PEEP during ventilation of two pigs on the iSAVE with closed-circuit ventilation. (**A**) Summary of ventilatory and respiratory parameters. Mean ± SD was calculated from 300 breathing cycles. No significant differences were found between ventilation with and without differential PEEP (homoscedastic two-tailed *t* test, *P* > 0.05). (**B**) Pressure, flow, and volume waveforms for the two animals. Pig A (blue) and pig B (black) were ventilated with PEEP of 5 and 10 cmH_2_O, respectively.

In the third stage, we ventilated the two animals with differential PEEPs (Pig A: PEEP = 5 cmH_2_O, Pig B: PEEP = 10 cmH_2_O, Fig. 4, A and B) using iSAVE on a closed-circuit ventilator. During ventilation, we simulated several practically challenging scenarios including: (i) Adding an animal to the iSAVE circuit; (ii) quickly removing an animal from the circuit without leaking the airflow (containing isoflurane) into the atmosphere; and (iii) adjusting ventilation parameters as the animal’s respiratory mechanics changed. In all cases, iSAVE enabled stable management of ventilation.

## DISCUSSION

In this study, we demonstrate how the iSAVE enables individualized management of ventilation using valves, sensors, and alarms. Through benchtop and in vivo testing, we demonstrate that we can not only individualize tidal volume and PEEP, but also rebalance ventilation to accommodate changes in respiratory mechanics in one channel that could otherwise jeopardize flow to a second connected channel. The data show that the system can support the flows and pressures required to manage the ventilation of lungs with properties of ARDS for two individuals over several hours. We tested several clinical scenarios associated with multiplexing ventilation to address alarm mechanisms, connection and disconnection processes, mitigation of contamination risk, and measurement of pulmonary mechanics. Moreover, the stability of tidal volume, PEEP, respiratory mechanics (resistance and compliance) and gas exchange (SpO_2_, EtCO_2_), as indicated by low standard deviation values during the ventilation of two pigs, suggest that it may be possible to integrate iSAVE in a protective ventilation strategy.

The iSAVE’s pressure release valve, flow control valve, one-way valve, pressure sensor, and CO_2_ sensor are commercially available medical-grade parts commonly found in hospitals, lowering the barrier to implementation. In the event of shortages, these parts could be procured from plumbing and ventilation departments in hardware stores and autoclaved for sterile usage. If standard adaptors do not interface these parts, adaptors can be made from standard piping or three-dimensional printing. As specified, the iSAVE permits individualized ventilation through alteration in tidal volume for two channels. Future iterations could incorporate closed-loop control, directly modulating flow according to flow meter readings and valves to allow for independent control of RR and FiO_2_ in each patient circuit. For ventilators requiring closed-loop flow control, standard flow sensors can be placed in line and rerouted through stopcocks to enable normal functioning of the ventilator’s self-check/calibration mechanisms (fig. S9). In the envisioned setup, individual patient monitors (such as the Philips NM3) would be used to set the initial conditions of the valves, perform periodic checks, and determine changes in the circuit. Standard humidification devices for airstreams would be connected in series with the iSAVE on the inspiratory channel. In addition to the components described here, a whistle ring could be added to the pressure release valve to provide an auditory alarm when high pressure develops in one segment (fig. S10). Independent monitoring mechanisms should also be developed.

Although we tested scenarios involving only two individuals and splitting among two channels under preclinical conditions, in theory the iSAVE should be able to accommodate more than two channels. Our testing thus far has provided a proof of concept for the iSAVE using a single ventilator with two individuals (1:2 ratio). Ventilator volume can be linearly divided to additional patients. Thus, the maximal capacity of this system is defined by:

Capacity = *V*_T,Ventilator_(*V*_T,Patient 1_+*V*_T,Patient 2_+*V*_T,Patient 3_+…) Eq. 1

Since most ICU ventilators provide up to 2500 mL, it is estimated that at least 6 individuals could be simultaneously ventilated. However, physical and practical challenges will limit implementation, particularly in the ICU setting. Dead space, the volume of air that doesn’t participate in gas exchange, accumulated in tubing cannot be greater than the lowest individual tidal volume required. Further, with the addition of more than two patients to one ventilator circuit, determining flow changes and titration will entail a more involved process, requiring monitors for each patient. In the case of *n* patients, data from *n-1* circuits would be required to make adjustments. If the supply of respiratory monitors is constrained, this limitation could potentially be overcome by sharing monitors among patients and using monitors to perform frequent checks.

Based on the iSAVE’s individualization capacity, matching criteria for patients may not need to be as stringent as other protocols designed to multiplex ventilation without individualized control (*10*). Nevertheless, certain ventilatory parameters (RR, FiO_2_, I:E) will remain shared amongst patients. Additionally, patients need to remain sedated (and/or paralyzed) to prevent spontaneous breathing, which would lead to asynchrony in the system. Thus, patients should be matched as closely as possible in terms of degree of illness and ventilatory needs to optimize functioning. A list of recommendations for patient stratification is provided in table S5. These guidelines were derived from the highest bounds of variance tolerable by the iSAVE from our in vitro and preclinical testing, with a safety factor of 2-3, in combination with feedback from standardized treatment protocols (*14*). Other considerations include hemodynamic stability, anticipated invasive ventilation time, proning, co-infection, and the logistics of space allocation for patients. Ideally, patients would be ventilated in negative pressure rooms, with their heads placed as close as possible to the ventilator. Recovering patients would need to be transitioned to an individual ventilator when spontaneous breathing becomes viable (*11*).

This study only tested the iSAVE on test lungs and in a small number of large animals without active disease pathology. Further, ventilation was performed for a short period of time in vivo to serve as a proof of concept. Our overall goal is to ensure that the iSAVE can be reliably implemented and used across intensive care clinical settings to address ventilator shortages. To this end, further steps must be taken to address current limitations prior to clinical implementation. Due to differences in performance characteristics (mechanism of pressure/flow monitoring) of ICU ventilators, this approach must be tested across a range of ventilators. The iSAVE must be evaluated in conditions reflecting the real-life variability of intensive care practice. The procurement and sterilization processes for non-standard components must be addressed. Toward facilitating this evaluation by our team and others, we have assembled a list of components, instructions for assembly, links to three-dimensional printable adaptors (barb, push-to-connect, or luer lock fittings), and clinical consideration guidelines (https://i-save.mit.edu/). Modules to train personnel in using this system should also be developed. Although this system mitigates several challenges associated with splitting ventilation, it has several drawbacks, unknown limitations, and does not fully address safety concerns associated with this and other approaches for ventilator splitting. Ventilator sharing is strongly discouraged by critical care practitioners due to safety concerns. In a setting of severe shortage of ventilators, sharing a ventilator among two patients could potentially mitigate the need to triage which patients receive ventilatory support and which do not. This may present societal value in comparison to potentially saving or prioritizing one life. Further ethical and policy-based discussions are necessary prior to implementation. The authors caution that the iSAVE approach is not a standard of clinical care and that this is a preclinical study. Rigorous further testing and validation of this approach is necessary before implementation could be considered under the Emergency Use Authorization issued by the U.S. Food and Drug Administration (*13*). The techniques and approaches described in this preclinical study do not represent any recommendation or alteration in the recommended use of devices that were studied in this article.

Here we have demonstrated that the iSAVE provides a solution for expansion of respiratory support using readily available medical-grade materials and exiting ventilators. The iSAVE can at least double existing ventilator capacity while retaining personalized ventilation settings for two individuals. Healthcare systems worldwide could potentially benefit from this system as they strive to care for the increasing volume of patients with ARDS associated with COVID-19 infection.

## MATERIALS AND METHODS

### Study design

The goal of this study was to design and validate a system using medical-grade filters, valves, and sensors to provide individualized ventilation using one ventilator shared among at least two subjects. We performed benchtop testing of the iSAVE using linear test lungs to ensure its ability to provide differential ventilation, accommodate for changes in respiratory mechanics, and manage acute changes to patient status. We also evaluated issues of cross contamination, adding and removing subjects from the circuits, and circuit design as they related to specific ventilator models. We performed ventilation of one pig and one test lung, wherein we modulated the respiratory mechanics of each lung to test the management of rebalancing differential ventilation to both lungs. We then performed the simultaneous ventilation of two pigs to evaluate the iSAVE’s performance in an in vivo setting, simulating several clinical scenarios. Animal experiments were approved by the Committee on Animal Care at the Massachusetts Institute of Technology (MIT). No blinding or randomization was performed as this was a proof-of-concept study.

### Open-circuit ventilator assembly

A Philips Trilogy portable ventilator was used as a representative open-circuit ventilator for testing. Standard flex corrugated tubing (22 mm outer diameter) was used to connect two respiratory circuits, each consisting of a (i) ball valve (EKWB G ¼’’ Nickel, Microcenter), (ii) bacterial/viral filter (Main Flow Bacterial/Viral Filter, Teleflex Medical), (iii) pressure sensor (TD160D, Biopac Systems), (iv) airflow sensor (BSL Medium Airflow Transducer SS11LA and AFT 20, Biopac Systems), and (v) Whisper Swivel valve (passive exhalation port, Philips). This was connected to the expiratory side of a Y-piece to allow for the inclusion of a bacterial/viral filter before venting exhaled gas to the ambient. See the circuit diagram provided in Fig. 1A. The airflow and pressure sensors were connected to DA100C differential amplifiers with 1000x gain and processed through the MP160WSW signal processing unit (Biopac Systems) sampling at 2 kHz. Volume control mode was employed, and settings were adjusted to deliver the desired *V*_T_, RR, and PEEP. Artificial linear test lungs (IngMar Medical) were used for simulations with the open-circuit ventilator.

### Closed-circuit ventilator assembly

The iSAVE is designed to work with closed-circuit ventilators currently found in ICUs. To this end, the system was tested on a Philips VX850 ventilator, a Hamilton G5 ventilator, and Puritan Bennett 840 ventilator (fig. S1). These ventilators were chosen as they represent the most common brands used in the United States. Y-connectors were connected to the inspiratory and expiratory limbs of the ventilator to enable individual channels for each lung. In the inspiratory limbs, the following components were connected in series: (i) a bacterial/viral filter (Main Flow Bacterial/Viral Filter, Teleflex Medical), (ii) ball valve (EKWB G ¼’’ Nickel, Microcenter), (iii) one-way valve (a Threshold PEP positive expiratory pressure device, Philips, was used as a surrogate for one-way valves), (iv) pressure sensor, (v) flow sensor, and (vi) capnostat adaptor. In the expiratory limb, the following components were connected in series: (i) a bacterial/viral filter, (ii) a pressure release valve (PEEP valve), and (iii) an optional one-way valve prior to connection with the ventilator (Threshold PEP). Polytetrafluoroethylene tubing or rubber adapters were used to adapt the ball valves to the standard 2-mm outer diameter corrugated flex tubing. A dual adult lung simulator (Model 5600i, Michigan Instruments) was used to perform simulations with the closed-circuit ventilator. Pressure and flow were displayed and recorded by the Philips NM3 monitors and associated sensors, one for each channel. For ventilators requiring continuous flow measurements for calibration and closed-loop control, such as the Hamilton G5, the standard flow sensors can be reconfigured using stopcocks as elaborated in fig. S9.

### Setup and testing protocol

The following main ventilator settings were selected: (i) Volume control mode is selected with triggering turned off; (ii) RR is determined based on the minute ventilation needs for both sets of lungs; (iii) FiO_2_ is set to the desired value to be shared for both patients; (iv) I:E ratio is set with consideration of the tau (τ = RC) parameter of each patient, which must initially be estimated for each patient. The longer τ will dictate the expiratory window and prevent autoPEEP; and (v) the alarm for minute volume was set to the following lower and upper bounds [10% of RR(*V*_T1_ + *V*_T2_), 10% of RR(*V*_T1_ + *V*_T2_)]. Next, we checked for leaks and alarms by first testing the ability of the system to perform ventilation without leaks. We then disconnected a lung to ensure the activation of standard alarms on the ventilator. To produce variable tidal volumes, starting with both flow valves fully open, we measured the flow and pressure delivered to each lung. Then, we gradually closed one of the valves, measuring the distribution of flow, to map the range of volume distribution capable of the system. To maintain *V*_T_ after static changes to compliance and resistance: We modulated the compliance and/or resistance of one test lung; measured the effect of the intervention; and titrated the valve until the baseline parameters were reached. Resistors (Rp5, Rp20, Rp50, and Rp500 Michigan Instruments) were used for all benchtop testing. We performed these tests with baseline parameters set to those of (i) a healthy lung (*C* = 50 cmH_2_O, *R* = Rp5) and (ii) a lung with ARDS (*C* = 20 cmH_2_O, *R* = Rp5-50). The flow valve was the main variable we modulated but, in some cases, the ventilator’s tidal volume or inspiration:expiration ratio was also adjusted. These cases are specifically mentioned in the results section. Last, to maintain desired ventilation after dynamic changes to compliance and resistance, we simulated acute changes by disconnecting or clamping tubes and monitoring the ventilator’s response. The flow valves were also titrated to return the circuit to its baseline.

### Cross-contamination testing

5 mL of Trypan Blue (0.4%, 15250061, Thermo Fisher) was placed in a nebulizer attached to the patient inflow/outflow segment of one of two circuits containing a test lung. Air from a separate line was used to nebulize droplet particles into the main tubing. We then performed tests ranging the following parameters, including the maximal combination designed to create the most turbulent flow conditions: valves: fully open to fully closed: airflow pressure used to nebulize: 1-2800 cmH_2_O, duration of nebulization: 1 – 10 min, *V*_T_ setting: 100-900 mL per lung, PEEP: 2 – 10 cmH_2_O, and RR: 2 – 30 bpm. After each test, filters were manually inspected for contamination and a wipe test was performed in each of the segments of tubing. Each wipe was then incubated in diH_2_O for 5 min. 1 mL from each sample was used to perform UV-vis absorption spectroscopy at a wavelength of 580 nm. Each sample was measured in triplicate.

### In vivo testing

The first experiment was performed as part of a terminal procedure on a 70 kg female swine (n = 1). The iSAVE was connected to a veterinary anesthesia ventilator (Model 200IE, Hallowell EMC) delivering 2% isoflurane in oxygen. One inspiratory circuit was connected to the anesthesia machine delivering gas to the animal while the other inspiratory circuit was connected to an artificial linear test lung (IngMar Medical). Pressure and flow measurements were recorded on the inspiratory limb of the animal. A VetTrends Vital Signs Monitor was utilized to measure SpO_2_, EtCO_2_, RR, and other physiological parameters. 600 mL of *V*_T_ were equally distributed between the animal and test lung. A respiratory rate between 18-20/min was set on the ventilator. We carried out the same tests outlined in the benchtop testing protocol, modulating the parameters of the test lung to validate the capabilities of the iSAVE to restore the system to baseline. After the animal was euthanized, to acutely change the compliance of the pig’s lung we used an endoscope (Pentax) to deliver 750 mL of phosphate buffered saline (PBS, Sigma Aldrich) into the left and right bronchi. Ventilation was performed and the valves were titrated to achieve desired flow parameters.

In the second experiment, one 74 kg and one 88 kg female swine were used in a survival approach. These pigs were sedated with 0.25 mg/kg (5 mg/mL) midazolam and 0.03 mg/kg (0.5 mg/mL) dexmedetomidine. The experiment was divided into three stages: Individual ventilation of each animal, PEEP = 0 (stage 1); iSAVE ventilation (differential *V*_T,_ PEEP = 0) (stage 2); and iSAVE ventilation (differential *V*_T_, differential PEEP) (stage 3). Ventilation was performed with the Philips Respironics Esprit ventilator in series with the anesthesia machine. The target tidal volume for each animal was calculated at 10 mL/kg, although ventilation was optimized to maintain SpO_2_ > 94% and EtCO_2_ ~ 30-35 mmHg in all stages. The PEEP setting on the main ventilator was set to 0 cmH_2_O. After 30 min of stable ventilation, arterial blood gases were measured using an i-STAT handheld blood analyzer (Abbott). Respiratory flow, pressure and volume were continuously logged using the Philips NM3 monitors as well as Biopac systems described above. Reported values represent the mean and standard deviations of 300 breathing cycles. In stage 3, the PEEP settings were changed by adjusting the PEEP valve to enable the animals to be ventilated with differential PEEP values (Pig A: PEEP = 5 cmH_2_O, Pig B: PEEP = 10 cmH_2_O). Compliance and resistance were measured for each animal by performing an end-inspiratory pause of 1 s to ensure that the iSAVE enabled these measurements. The computed compliance and resistance values matched those which were measured on the monitor. The duration of each stage lasted about 45 min.

### Data and statistical analysis

Pressure and flow data from the Biopac system was analyzed using MATLAB (2018a). Flow data was integrated every respiratory cycle to derive the flow. In cases where pressure and flow data were not simultaneously obtained from both limbs of the circuit, data were derived based on the settings on the main ventilator and the measurements on one limb. Homoscedastic, two-tailed *t* tests were performed to assess significance on the data from the contamination testing with a threshold of *P* < 0.05. A homoscedastic, two-tailed, *t* test was used to compare the values from 300 breathing cycles between the two conditions for the in vivo study with a threshold of *P* < 0.05.

## SUPPLEMENTARY MATERIALS

stm.sciencemag.org/cgi/content/full/scitranslmed.abb9401/DC1 Fig. S1. Photographs of the iSAVE setup Fig. S2. Differential tidal volume and PEEP delivery on the iSAVE using an open-circuit ventilator and test lungs Fig. S3. Accommodation to changes in compliance of one test lung using the iSAVE and an open-circuit ventilator Fig. S4. Ventilation at high lung resistances Fig. S5. Ventilator alarm response to occlusion Fig. S6. Adding a test lung to the circuit Fig. S7. Cross-contamination validation using artificial lungs Fig. S8. Individualized management of ventilation using iSAVE on a pig lung and a test lung Fig. S9. Modification of sensing circuit for Hamilton G5 ventilator. Fig. S10. Whistle ring designs for two types of common pressure release (PEEP) valves Table S1. List of components required for the assembly of the iSAVE Table S2. Mechanical components used in the iSAVE and their readily available medical industry equivalents Table S3. Measurement of respiratory mechanics for iSAVE system using test lungs Table S4. Blood electrolytes and chemistry during ventilation in pigs Table S5. Stratification for patient matching
